# Relative Effectiveness of the MF59-Adjuvanted Influenza Vaccine Versus High-Dose Influenza Vaccine in Older Adults With Influenza Risk Factors During the 2019–2020 US Influenza Season

**DOI:** 10.1093/ofid/ofae459

**Published:** 2024-08-16

**Authors:** Mahrukh Imran, Carrie W Mills, Kimberly W McDermott, Alex Dean, Alina Bogdanov, Ian McGovern, Mendel D M Haag

**Affiliations:** Center for Outcomes Research & Epidemiology, CSL Seqirus, Kirkland, Quebec, Canada; Real World Evidence, Veradigm, Chicago, Illinois, USA; Real World Evidence, Veradigm, Chicago, Illinois, USA; Real World Evidence, Veradigm, Chicago, Illinois, USA; Real World Evidence, Veradigm, Chicago, Illinois, USA; Center for Outcomes Research & Epidemiology, CSL Seqirus, Waltham, Massachusetts, USA; Center for Outcomes Research & Epidemiology, CSL Seqirus, Amsterdam, The Netherlands

**Keywords:** comorbidity, hospitalization, influenza, older adults, vaccine effectiveness

## Abstract

**Background:**

This study estimated the relative vaccine effectiveness (rVE) of the MF59-adjuvanted trivalent influenza vaccine (aTIV) versus high-dose trivalent inactivated influenza vaccine (HD-TIV) for prevention of influenza-related medical encounters (IRMEs) during the 2019–2020 United States (US) influenza season stratified by the cumulative number of influenza risk factors. A secondary objective evaluated outpatient IRMEs and influenza- and pneumonia-related hospitalizations.

**Methods:**

This retrospective cohort study included US adults ≥65 years old vaccinated with aTIV or HD-TIV between 1 August 2019 and 31 January 2020. Electronic health records linked to claims were used to ascertain exposure, covariates, risk factors, and outcomes. Multivariable adjusted odds ratios (ORs) were derived using inverse probability of treatment–weighted samples to calculate rVEs independently for individuals with 0, ≥1, 1–2, or ≥3 risk factors.

**Results:**

The study included 1 115 725 aTIV and 2 561 718 HD-TIV recipients. For the primary outcome of any IRME, the analysis found comparable effectiveness between aTIV and HD-TIV (rVE, 5.2% [95% confidence interval {CI}, −5.9% to 15.1%]) among those with 0 risk factors, whereas aTIV was more effective than HD-TIV among patients with ≥1, 1–2, or ≥3 risk factors (12.5% [95% CI, 10.0%–15.0%], 18.4% [95% CI, 13.7%–22.9%], and 10.4% [7.4%–13.3%], respectively). The same trends were observed for the secondary outcomes.

**Conclusions:**

This study demonstrated comparable effectiveness of aTIV and HD-TIV among individuals with no identified risk factors and higher effectiveness of aTIV compared with HD-TIV in preventing any IRMEs, outpatient IRMEs, and influenza- or pneumonia-related hospitalizations among those with at least 1 or multiple high-risk factors in adults ≥65 years old.

## Teaser

This study demonstrates benefit of an MF59-adjuvanted trivalent influenza vaccine compared with a high-dose vaccine in prevention of influenza-related medical encounters among high-risk individuals ≥65 years of age and comparable effectiveness in patients with no influenza high-risk factors.

Between 2010 and 2020, the Centers for Disease Control and Prevention (CDC) estimated the annual influenza burden to include 4.3–21 million medical visits, 140 000–710 000 hospitalizations, and 12 000–52 000 deaths in the United States (US) [1]. Adults ≥65 years of age are at increased risk of serious complications from influenza, including hospitalization and death, and these risks increase with age, the presence of certain chronic health conditions, and other factors [2–4]. Infection with influenza can also result in extrapulmonary complications, such as cardiovascular events, secondary bacterial infections, and exacerbations of chronic underlying conditions [5–7].

In addition to older adults being at greater risk of severe influenza and morbidity, standard-dose nonadjuvanted influenza vaccines are often less effective in older adults [8–10] due to immunosenescence, the progressive decline and dysregulation of the immune system associated with aging [11–13]. Two vaccine formulations specifically designed for use in this at-risk population are the MF59-adjuvanted trivalent influenza vaccine (aTIV) and the high-dose trivalent influenza vaccine (HD-TIV) [14, 15]. The relative vaccine effectiveness (rVE) of these formulations has been extensively compared among older adults, and they are generally found to have similar effectiveness [16, 17]. One study of the 2017–2018 and 2018–2019 influenza seasons found comparable effectiveness between aTIV and HD-TIV­­ among older adults with at least 1 underlying health condition that is known to increase a person's risk of experiencing severe influenza [18]; however, there is limited research on how an increasing number of risk factors may impact the relative effectiveness of these formulations among older adults.

As chronic health conditions are common among people over 65 [19], and the accumulation of multiple concurrent high-risk conditions is associated with increased rates of influenza-related hospitalizations [20], it is important to examine the effectiveness of vaccination among older adults with multiple risk factors. The primary objective of this study was to estimate the rVE of aTIV versus HD-TIV for the prevention of influenza-related medical encounters (IRMEs) stratified by the number of influenza risk factors that each patient had (ie, 0, ≥1, 1–2, ≥3). A secondary objective evaluated the rVE of aTIV versus HD-TIV for prevention of outpatient IRMEs specifically and influenza- or pneumonia-related hospitalizations stratified by the number of influenza risk factors in the same population.

## METHODS

### Study Design

This observational retrospective cohort study was conducted for the 2019–2020 influenza season among US residents aged ≥65 years.

### Study Period

We defined the influenza season as 29 September 2019 to 7 March 2020. The CDC defines the influenza surveillance season as epidemiologic week 40 through week 20 of the subsequent year, which corresponds to 29 September 2019 through 16 May 2020. We truncated the end of the observation period to avoid overlap with the coronavirus disease 2019 (COVID-19) pandemic, which affected both healthcare-seeking behavior and influenza circulation.

### Data Sources

The dataset used in the analysis was an integrated dataset of patient-level electronic health records (EHRs) from primary care and specialty clinics (Veradigm Network EHR), linked with pharmacy and medical claims data (Komodo Healthcare Map) for approximately 123 million individuals from all 50 US states. The integrated dataset provides comprehensive pharmaceutical, demographic, diagnostic, and healthcare utilization information and has been previously described in Boikos et al [21]. Both open and closed claims were utilized in this analysis.

### Ethical Compliance

This linked EHR and claims dataset has been certified as statistically de-identified through a formal determination by a qualified expert as defined in Section §164.514(b)(1) of the Health Insurance Portability and Accountability Act of 1996 (HIPAA) Privacy Rule.

The study was designed, implemented, and reported in accordance with Good Pharmacoepidemiological Practice, applicable local regulations, and the ethical principles laid down in the Declaration of Helsinki. All analyses reported were predefined in a study protocol prior to data analysis. The protocol was signed and dated by the study sponsor and the research organization that conducted the analysis prior to the generation of any results. Study results have been reported according to the Reporting of Studies Conducted using Observational Routinely Collected Health Data (RECORD) recommendations [22].

### Study Population

**Figure 1.**
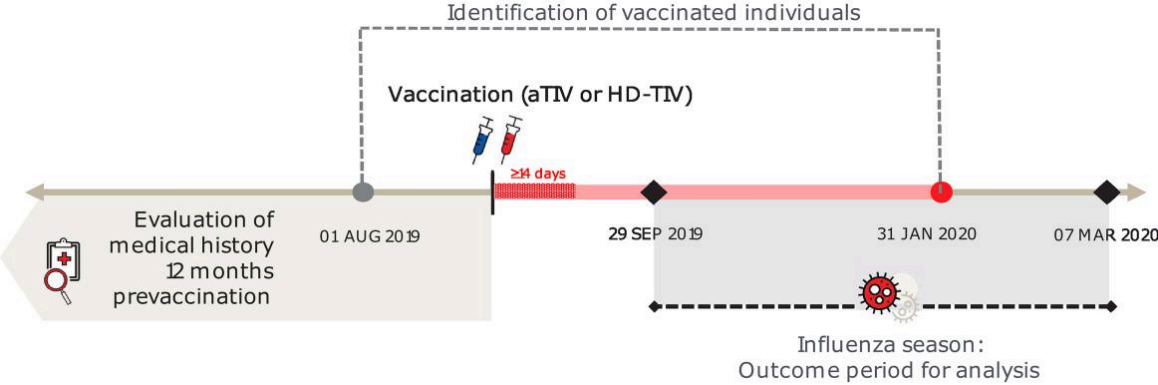
Schematic depiction of the observation period. Abbreviations: aTIV, adjuvanted trivalent inactivated influenza vaccine; HD-TIV, high-dose trivalent inactivated influenza vaccine.

The study population comprised US residents ≥65 years of age who received either aTIV or HD-TIV during the vaccination identification period (1 August 2019 through 31 January 2020; Figure 1) using the codes listed in Supplementary Table 1. The date of recorded vaccination was considered the index date. Eligible individuals were required to have a record in the EHR data within 12 months prior to the index date, as well as a claim at least 12 months prior to the index date and a claim after the end of the influenza season. Individuals were excluded if they had a record of any influenza vaccination between the end of the previous influenza season (19 May 2019) and the day before the start of the vaccination identification period, had >1 influenza vaccination administration between the beginning of the vaccination identification period and the end of the influenza season, had an IRME after the end of the previous influenza season and either before becoming fully vaccinated or before the start of the influenza season, or had missing sex or geographic information.

### Strata of Influenza Risk Factors

Individuals were stratified according to the presence of CDC-defined risk factors associated with an increased risk of complications due to influenza [2]. These CDC risk factors included asthma, neurologic and neurodevelopmental conditions, blood disorders, chronic lung disease, endocrine disorders, heart disease and related conditions, kidney diseases, liver disorders, metabolic disorders, obesity with a body mass index of ≥40 kg/m^2^, weakened immune system, and history of stroke. Medical conditions that belonged to the same clinical category (such as emphysema and chronic obstructive pulmonary disease) were counted as a single risk factor (chronic lung disease), whereas medical conditions belonging to different clinical categories (such as coronary artery disease and chronic kidney disease) were counted as multiple risk factors (heart disease and kidney disease). The codes defining risk factors are listed in Supplementary Table 1. Individuals were stratified according to the presence of 0 risk factors, any risk factors (≥1), or categories of 1–2, or ≥3 cumulative risk factors based on an a priori feasibility assessment.

### Exposure Ascertainment

We identified patients vaccinated with aTIV or HD-TIV during the vaccination identification period. Individuals were considered vaccinated 14 days after the index date to allow for the development of vaccine-specific immunity.

### Outcomes

The outcome for the primary objective was the occurrence of any IRME, using *International Classification of Diseases, 10th Edition, Clinical Modification* (*ICD-10-CM*) codes J09*, J10*, and J11* in any diagnosis position, as defined in the US Armed Forces Health Surveillance Center's validated diagnostic Code Set B [23]. The outcomes for the secondary objective were outpatient IRMEs and influenza- or pneumonia-related hospitalizations. Outpatient IRMEs were identified using the same diagnosis codes as the primary IRME outcome but limited to diagnoses associated with outpatient encounters. Influenza- or pneumonia-related hospitalizations were identified using *ICD-10-CM* codes J09*–J18* (Supplementary Table 1) in any diagnosis position. Outcomes were evaluated for each individual if they occurred >14 days after influenza vaccination and during the 2019–2020 influenza season as defined above.

### Covariates

Covariates, as defined in Supplementary Table 2, were measured in the 12 months prior to vaccination. Demographic characteristics included age, sex, race, ethnicity, and US geographic region. Baseline healthcare utilization metrics included the mean number of all-cause outpatient visits, inpatient admissions, and emergency room visits. Additional covariates included the week of vaccination, the Faurot frailty index score [24], and the presence of individual risk factors. Including individual risk factor categories in the model allowed for the adjustment of imbalances between the types of risk factors among individuals who received aTIV versus HD-TIV.

### Statistical Methods—Primary and Secondary Objectives

Baseline differences in covariates between aTIV and HD-TIV exposure cohorts were assessed for each risk factor stratum (ie, 0, ≥1, 1–2, ≥3) using standardized mean differences (SMDs) [25], with an absolute value of ≤0.1 indicating a negligible difference.

The main analyses determined the rVE for aTIV versus HD-TIV for both the primary and secondary outcomes for each stratum of cumulative number of risk factors. Odds ratios (ORs) were calculated using logistic regression with vaccine type as the independent variable. rVEs were calculated as 100 × (1 − OR) and reported with 95% confidence intervals (CIs). A doubly robust approach was used, which included the inverse probability of treatment weighting (IPTW) followed by multivariable adjustment of the inverse probability of treatment (IPT) weighted sample using logistic regression models that included all study covariates [26]. Both the propensity score model used to derive IPT weights and the multivariable adjustment included all covariates. These methods have been previously described by Imran et al [27]. Weighting and adjusting were conducted independently for each risk factor stratum.

Missing demographic variables were reported as “not reported” or “missing.” Analyses were conducted using SQL and SAS software (version 9.4).

### Statistical Methods—Post Hoc Analyses

Two post hoc analyses were conducted to validate the main results by assessing if residual confounding may have contributed to the observed results: (1) a descriptive analysis of the time between exposure and outcome and (2) an analysis of negative control outcomes (NCOs).

The first post hoc analysis described the time between vaccination and each outcome of interest by vaccine cohort within each risk factor stratum to evaluate whether outcomes occurred with similar temporality for each vaccine cohort. The median number of days (interquartile range) from vaccination to outcome was calculated. Additionally, outcome counts for vaccine cohorts by week of outcome were generated and graphed to visually assess potential differences.

The NCOs served as indicators of potential unmeasured or residual bias. The NCO analysis first examined the distribution of NCOs between the aTIV and HD-TIV cohorts within each cumulative risk factor stratum. Five negative control conditions were examined: appendicitis, cataracts, eyelid disorders, ingrown nails, and lipomas. These conditions were selected as they are hypothesized to be associated with health-seeking behavior and due to their anticipated lack of association with influenza vaccine exposure and influenza. Some of these NCOs have been evaluated in past influenza vaccine effectiveness studies [28].

Outcomes included encounters in any setting and in any diagnosis position. SMDs were generated to assess differences in the incidence of each NCO between the exposure cohorts prior to and following IPTW. Next, the rVE of aTIV versus HD-TIV in preventing each of the NCOs in any setting for each high-risk factor stratum was estimated to evaluate the potential effect of unmeasured or residual confounding on effect estimates. The same doubly robust weighting and adjustment methodology was used as the main analysis.

## RESULTS

### Study Subjects

**Figure 2.**
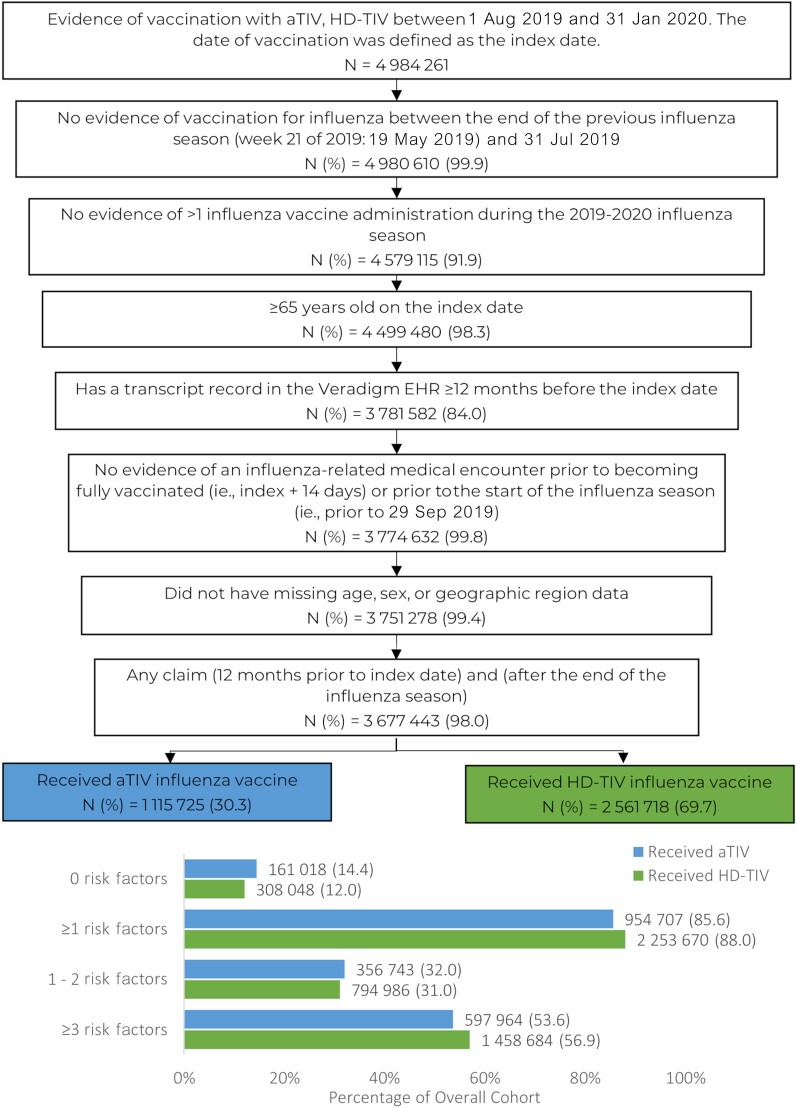
Patient selection for individuals with adjusted or high-dose trivalent inactivated influenza vaccines, and number of influenza risk factors. Abbreviations: aTIV, adjuvanted trivalent inactivated influenza vaccine; EHR, electronic health record; HD-TIV, high-dose trivalent inactivated influenza vaccine.

The overall study cohort consisted of 3 677 443 vaccinated individuals ≥65 years of age, of whom 1 115 725 (30.3%) received aTIV and 2 561 718 (69.7%) received HD-TIV (Figure 2). The proportion of aTIV and HD-TIV recipients in each risk factor stratum was similar, with 14.4 versus 12.0% in the 0 risk factor stratum, 85.6 versus 88.0% in the any (≥1) risk factor stratum and specifically 32.0 versus 31.0% in the 1–2 risk factor stratum, and 53.6 versus 56.9% in the ≥3 risk factor stratum.

Baseline demographic and clinical characteristics of individuals in each risk factor stratum for those vaccinated with aTIV or HD-TIV, along with SMDs before and after weighting, are reported in Supplementary Tables 3–10. The demographic and clinical characteristics were well-balanced in all risk factor strata after IPTW, with the exception of vaccination in the month of August, as aTIV recipients tended to get the vaccine earlier in the vaccination period. Cohort sizes before and after weighting are reported in Supplementary Table 11. Heart disease, metabolic disorders, and endocrine disorders were the most common risk factors regardless of the total number of risk factors (Supplementary Tables 12–14).

### Main Analyses

In all risk factor strata, 0.29%–0.89% of individuals in any vaccine cohort had an IRME, 0.27%–0.73% had an outpatient IRME, and 0.13%–1.19% had an influenza- or pneumonia-related hospitalization. The unweighted and weighted exposure-outcome results for the primary and secondary objectives are reported in Supplementary Tables 15 and 16. The unweighted and unadjusted rVEs are reported in Supplementary Table 17.

**Figure 3.**
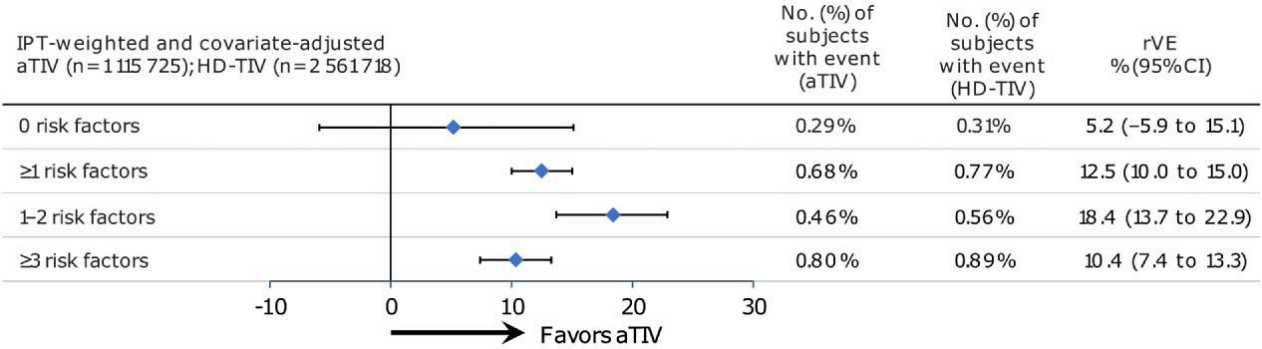
The doubly robust weighted and adjusted relative vaccine effectiveness for the primary outcome of any influenza-related medical encounter. Abbreviations: aTIV, adjuvanted trivalent inactivated influenza vaccine; CI, confidence interval; HD-TIV, high-dose trivalent inactivated influenza vaccine; IPT, inverse probability of treatment; rVE, relative vaccine effectiveness.

Among older adults with risk factors for complications due to influenza, aTIV was more effective than HD-TIV at preventing any IRME (Figure 3). The doubly robust rVEs (95% CI) for aTIV versus HD-TIV were 12.5% (10.0%–15.0%) for individuals with ≥1 risk factor, 18.4% (13.7%–22.9%) for individuals with 1–2 risk factors, and 10.4% (7.4%–13.3%) for individuals with ≥3 risk factors. Among those with 0 risk factors, the CI for the rVE estimate included the null (5.2% [−5.9% to 15.1%]), suggesting comparability in the effectiveness of the 2 vaccines for this stratum of older adults.

**Figure 4.**
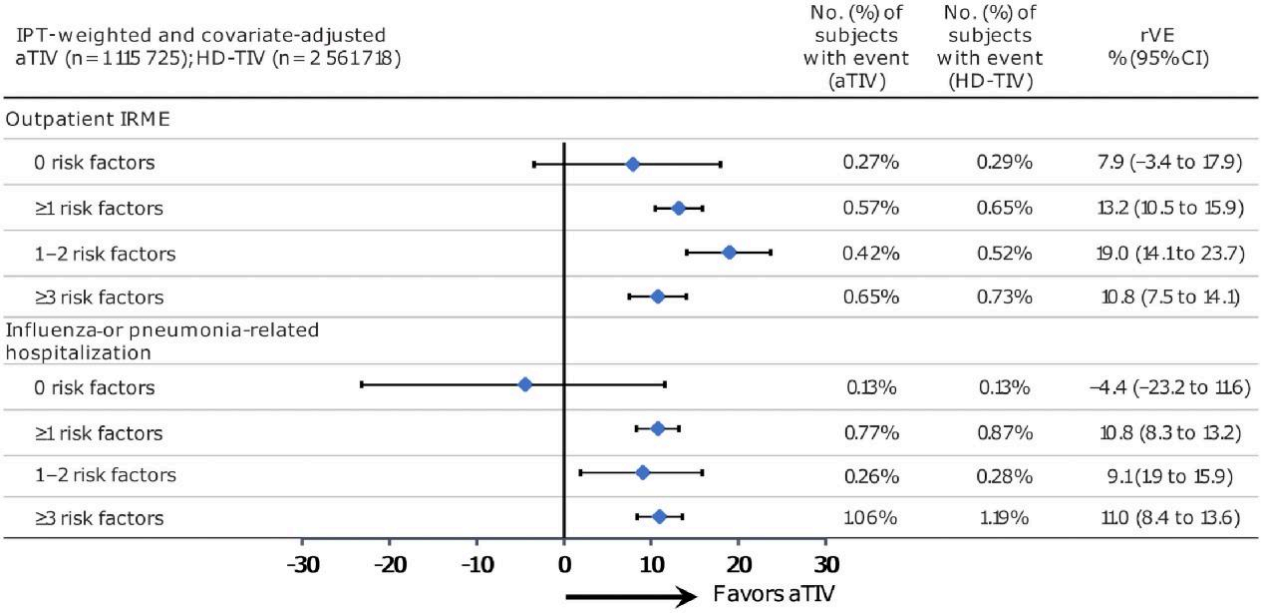
The doubly robust weighted and adjusted relative vaccine effectiveness for secondary outcomes. Abbreviations: aTIV, adjuvanted trivalent inactivated influenza vaccine; CI, confidence interval; HD-TIV, high-dose trivalent inactivated influenza vaccine; IPT, inverse probability of treatment; IRME, influenza-related medical encounter; rVE, relative vaccine effectiveness.

Observations for the secondary outcomes were similar to the primary outcome. aTIV was more effective than HD-TIV at preventing outpatient IRMEs and influenza- or pneumonia-related hospitalizations among older adults with risk factors (Figure 4). For outpatient IRMEs, the rVEs (95% CI) for aTIV versus HD-TIV were 13.2% (10.5%–15.9%) for individuals with ≥1 risk factor, 19.0% (14.1%–23.7%) for individuals with 1–2 risk factors, and 10.8% (7.5%–14.1%) for individuals with ≥3 risk factors. For influenza- or pneumonia-related hospitalizations, the rVEs (95% CI) for aTIV versus HD-TIV were 10.8% (8.3%–13.2%) for individuals with ≥1 risk factor, 9.1% (1.9%–15.9%) for individuals with 1–2 risk factors, and 11.0% (8.4%–13.6%) for individuals with ≥3 risk factors. As with the primary objective, among those with 0 risk factors, the CI for the rVE estimate for both secondary objectives included the null, suggesting comparability in the effectiveness of the 2 vaccines for this stratum.

### Post Hoc Analyses

As previous research has observed comparable vaccine effectiveness between aTIV and HD-TIV, this study sought to understand if the higher effectiveness observed for aTIV could be due to timing of vaccination or residual confounding. The first post hoc analysis suggested that across high-risk factor strata, the median number of days between vaccination and outcome was approximately the same for those in the aTIV and HD-TIV exposure cohorts (Supplementary Table 18). In addition, the calendar distribution of outcomes was similar between vaccine groups (Supplementary Figures 1–3).

Across all high-risk factor strata, the incidence of NCO analysis was balanced pre- and post-weighting (ie, |SMDs| **≤**0.1) between patients who received aTIV and patients who received HD-TIV (Table 1). The rVEs for the NCOs can be found in Supplementary Tables 19 and 20. The CIs of the doubly robust rVEs included the null value in all risk factor strata for the outcomes of appendicitis and lipomas and included the null value in the any (≥1) risk factor stratum for the outcomes of cataracts and in the ≥3 risk factor stratum for the outcomes of cataracts and eyelid disorders. In all NCOs where the CI did not include the null value, the rVE was negative, suggesting that any potential residual bias would have favored HD-TIV. No patterns were observed across the NCOs that would suggest residual confounding factors to be biased toward favoring aTIV.

**Table 1.** Distribution of Negative Control Outcomes

Outcome	aTIV		HD-TIV		SMD	
	(n = 1 115 725)		(n = 2 561 718)			
	NO.	%	NO.	%	Unweighted	IPT Weighted
0 Risk factors	161 018	100.0%	308 048	100.0%	…	…
Appendicitis	49	0.0%	71	0.0%	0.005	0.006
Cataracts	10 683	6.6%	18 146	5.9%	0.031	0.012
Eyelid disorders	2861	1.8%	4663	1.5%	0.021	0.009
Ingrown nails	706	0.4%	1102	0.4%	0.013	0.010
Lipomas	281	0.2%	498	0.2%	0.003	0.001
≥1 Risk factor	954 707	100.0%	2 253 670	100.0%	…	…
Appendicitis	400	0.0%	895	0.0%	0.001	0.000
Cataracts	89 920	9.4%	201 779	9.0%	0.016	0.001
Eyelid disorders	28 480	3.0%	61 735	2.7%	0.015	0.002
Ingrown nails	14 154	1.5%	31 814	1.4%	0.006	0.006
Lipomas	3477	0.4%	7872	0.3%	0.002	0.001
1–2 Risk factors	356 743	100.0%	794 986	100.0%	…	…
Appendicitis	144	0.0%	274	0.0%	0.003	0.003
Cataracts	31 490	8.8%	65 295	8.2%	0.022	0.006
Eyelid disorders	9456	2.7%	18 715	2.4%	0.019	0.007
Ingrown nails	3227	0.9%	6515	0.8%	0.009	0.007
Lipomas	1108	0.3%	2374	0.3%	0.002	0.000
≥3 Risk factors	597 964	100.0%	1 458 684	100.0%	…	…
Appendicitis	256	0.0%	621	0.0%	0.000	0.001
Cataracts	58 430	9.8%	136 484	9.4%	0.014	0.002
Eyelid disorders	19 024	3.2%	43 020	2.9%	0.013	0.000
Ingrown nails	10 927	1.8%	25 299	1.7%	0.007	0.005
Lipomas	2369	0.4%	5498	0.4%	0.003	0.001

Abbreviations: aTIV, adjuvanted trivalent inactivated influenza vaccine; HD-TIV, high-dose trivalent inactivated influenza vaccine; IPT, inverse probability of treatment; SMD, standardized mean difference.

## DISCUSSION

In this analysis of the 2019–2020 influenza season, vaccination with aTIV, compared to HD-TIV, was associated with fewer IRMEs among adults at least 65 years old who had any risk factors (≥1) or categories of 1–2 or ≥3 cumulative risk factors for severe influenza. No difference in vaccine effectiveness was observed among older adults with 0 risk factors for the primary outcome. The same trends were observed for the secondary outcomes of outpatient IRME and influenza- or pneumonia-related hospitalization.

Most prior studies have shown comparability in the effectiveness of aTIV versus HD-TIV in preventing IRMEs with or without pneumonia in adults aged ≥65 years, including among adults with any risk factors [18, 29–33]. For example, a meta-analysis of studies by Coleman et al reported a pooled estimate of the rVE of aTIV versus HD-TIV of 3.2% (95% CI, −2.5% to 8.9%), suggesting comparable effectiveness of these 2 vaccines when assessed among all older adults [17]. Another meta-analysis by Domnich and de Waure also concluded that aTIV and HD-TIV have similar effectiveness in preventing seasonal influenza [16]. Similarly, the only prior study to examine rVE in older adults at high risk of influenza complications found no difference in the effectiveness of aTIV versus HD-TIV at preventing IRMEs in the 2017–2018 and 2018–2019 influenza seasons [18]. In this study, we sought to repeat the prior analysis of any risk factors in the 2019–2020 season and look more closely at individuals with multiple risk factors, as our recent analysis found that the number of risk factors correlated with odds of influenza hospitalization [20].

The post hoc analyses did not indicate that the benefit of aTIV observed in this study could be explained by the time since vaccination. Similarly, the NCO analysis did not suggest residual confounding favoring aTIV that could explain the benefit of aTIV over HD-TIV observed for those with 1 or more risk factors.

Both aTIV and HD-TIV produce a strong immune response among older adults despite the onset of immunosenescence in this population [34, 35]. In addition, the MF59 adjuvant used in aTIV has been shown to broaden the immune response, resulting in protection against variant strains not included in the vaccine [36]. This broader protection may account for the higher vaccine effectiveness in the most vulnerable population observed in this study and may also explain why, in some analyses, the rVE of the vaccines is comparable. Variability in rVE is expected depending on the epidemiological characteristics of a specific influenza season and the populations being studied. In the 2019–2020 season, within influenza A(H1N1)pdm09 HA clade 6B.1A, which includes the vaccine strain, further genetic diversification was observed [37]. The inclusion of an adjuvant would be expected to confer a benefit since the higher dose of antigen is not expected to improve effectiveness against the drifted strain. Additional research to further investigate these findings could provide a deeper understanding of how the immune response generated by aTIV, attributed to the MF59 adjuvant, impacts protection against variant strains and contributes to overall vaccine effectiveness across populations with different risk profiles.

No difference was seen among individuals with 0 risk factors. The absence of risk factors among a subset of older adults suggests a generally healthier population. Healthier individuals may inherently mount a more robust immune response to vaccination, potentially leading to higher absolute vaccine effectiveness (aVE) and comparable performance of both vaccines. However, it is important to note that we did not estimate aVE of aTIV and HD-TIV, precluding definitive conclusions about the factors impacting outcomes in this risk factor stratum. Further research, including exploration of aVE, may yield a more comprehensive understanding of vaccine performance in older adults with no risk factors.

There are several limitations to this analysis, including that the nature of individuals’ use of the US health system and the heterogeneity inherent in that system is a potential source of bias. Patient utilization of healthcare resources is intermittent or opportunistic; therefore, the amount and quality of data available on individuals may vary, and these could potentially be differential between vaccine groups. Furthermore, vaccination was not randomly assigned, and unmeasured confounding might bias estimates. We conducted an NCO analysis to determine whether any residual bias exists. Influenza-related events are not necessarily laboratory-confirmed, which might lead to outcome misclassification but is not expected to be differential between the vaccine groups. The methodology used in this study leverages available data, but EHR and claims data sources may be incomplete for some fields (eg, race and ethnicity) and do not include individual or contextual socioeconomic data that could inform health-seeking behavior.

This study also has several strengths, including the use of a large dataset integrating sources of patient information, which allowed for the evaluation of the impact of most CDC risk factors that are associated with increased risk of complications due to influenza, except race and being institutionalized. The inclusion of both EHR and claims data increases the likelihood that most—if not all—medical interventions and diagnoses necessary for identifying exposure and outcomes are captured within the study dataset. Furthermore, the variety and completeness of data allowed for the adjustment of well-established confounders. Exposure, outcome, and covariate information were ascertained retrospectively from patient records in the same manner for all exposure cohorts, limiting the possibility of differential misclassification of these elements. Finally, the use of a doubly robust IPTW methodology has the advantages of maximizing the retention of study patients and reducing the potential for estimate bias [38, 39].

## CONCLUSIONS

In the 2019–2020 influenza season, aTIV was more effective than HD-TIV in reducing any IRMEs, outpatient IRMEs, and influenza- or pneumonia-related hospitalizations in adults ≥65 years of age with cumulative risk factors. No statistically significant differences were observed among patients with no identified risk factors.

## Supplementary Material

ofae459_Supplementary_Data

## Supplementary Data

Supplementary materials are available at *Open Forum Infectious Diseases* online. Consisting of data provided by the authors to benefit the reader, the posted materials are not copyedited and are the sole responsibility of the authors, so questions or comments should be addressed to the corresponding author.

## Notes

***Acknowledgments.*** Jessamine Winer-Jones, an employee of Veradigm, provided medical writing support for this manuscript. This support was funded by CSL Seqirus.

***Author contributions.*** M. I., I. M., and M. D. M. H. contributed to study conceptualization. All authors contributed to study design, analysis, and interpretation. C. W. M., K. W. M., A. D., and A. B. contributed to data collection and data processing. M. I. and I. M. led development of the first draft of the manuscript. All authors revised the paper critically, made substantive intellectual contributions to the development of this manuscript, and approved the final version.

***Patient consent.*** The linked EHR and claims dataset used in this study has been certified as statistically de-identified through a formal determination by a qualified expert as defined in Section §164.514(b)(1) of the HIPAA Privacy Rule. Because this study used only de-identified patient records, it is therefore no longer subject to the HIPAA Privacy Rule and is therefore exempt from institutional review board approval and for obtaining informed consent according to US law.

***Data availability.*** The data that support the findings of this study were used under license from Veradigm and Komodo Health. Due to data use agreements and its proprietary nature, restrictions apply regarding the availability of the data. Further information is available from the corresponding author.

***Financial support.*** This work was supported by CSL Seqirus.

***Potential conflicts of interest*.** M. I., I. M., and M. D. M. H. are employees of CSL Seqirus. C. W. M., K. W. M., A. D., and A. B. work for Veradigm, a company that was contracted by CSL Seqirus and received a research contract to conduct this study with and on behalf of CSL Seqirus.
