# Supplementary material for: Relative Effectiveness of the MF59-Adjuvanted Influenza Vaccine Versus High-Dose Influenza Vaccine in Older Adults With Influenza Risk Factors During the 2019–2020 US Influenza Season
Source: Open Forum Infect Dis. 2024 Aug 16;11(8):ofae459. doi: 10.1093/ofid/ofae459 (PMC11337123; doi:10.1093/ofid/ofae459)

Supplementary Files for OFID Article

Title: Relative Effectiveness of the MF59®-Adjuvanted Influenza Vaccine vs High-Dose Influenza Vaccine in Older Adults with Influenza Risk Factors During the 2019-2020 U.S. Influenza Season

Authors: Mahrukh Imran[1], Carrie W. Mills[2], Kimberly W. McDermott[2], Alex Dean[2], Alina Bogdanov[2], Ian McGovern[3], Mendel D.M. Haag[4]

Affiliation: [1]CSL Seqirus, Kirkland, Quebec, Canada; [2]Veradigm, Chicago, IL, USA; [3]CSL Seqirus, Waltham, MA, USA; [4]CSL Seqirus, Amsterdam, The Netherlands

Table S1. Code Sets for Influenza Vaccines, Outcome Measures, and Risk Factors

Table S2. Covariate Definitions

Table S3. Demographic Characteristics and SMDs Before and After IPTW in the 2019–2020 Influenza Season (September 29, 2019 – March 7, 2020) by Vaccination Cohort Among Patients with 0 Risk Factors

Table S4. Demographic Characteristics and SMDs Before and After IPTW in the 2019–2020 Influenza Season (September 29, 2019 – March 7, 2020) by Vaccination Cohort Among Patients with ≥1 Risk Factors

Table S5. Demographic Characteristics and SMDs Before and After IPTW in the 2019–2020 Influenza Season (September 29, 2019 – March 7, 2020) by Vaccination Cohort Among Patients 1–2 Risk Factors

Table S6. Demographic Characteristics and SMDs Before and After IPTW in the 2019–2020 Influenza Season (September 29, 2019 – March 7, 2020) by Vaccination Cohort Among Patients with ≥3 Risk Factors

Table S7. Clinical Characteristics and SMDs Before and After IPTW in the 2019–2020 Influenza Season (September 29, 2019 – March 7, 2020) by Vaccination Cohort Among Patients with 0 Risk Factors

Table S8. Clinical Characteristics and SMDs Before and After IPTW in the 2019–2020 Influenza Season (September 29, 2019 – March 7, 2020) by Vaccination Cohort Among Patients with ≥1 Risk Factors

Table S9. Clinical Characteristics and SMDs Before and After IPTW in the 2019–2020 Influenza Season (September 29, 2019 – March 7, 2020) by Vaccination Cohort Among Patients with 1–2 Risk Factors

Table S10. Clinical Characteristics and SMDs Before and After IPTW in the 2019–2020 Influenza Season (September 29, 2019 – March 7, 2020) by Vaccination Cohort Among Patients with ≥3 Risk Factors

Table S11. Sample Size Pre- and Post-weighting

Table S12. Most Common Factors Among Patients with 1 Risk Factor

Table S13. Most Common Conditions Among Patients with 2 Risk Factors

Table S14. Most Common Conditions Among Patients with 3 Risk Factors

Table S15. Unweighted Exposure-Outcome Results Table

Table S16. Weighted Exposure-Outcome Results Table

Table S17. Unweighted and Unadjusted rVEs for Any IRME, Outpatient IRME, and Influenza- or Pneumonia-related Hospitalization in the 2019–2020 Influenza Season (September 30, 2019 – March 7, 2020) for Patients Aged 65+

Table S18. Days in Time from Vaccination to Outcome; weighted

Table S19. Unweighted and Unadjusted rVEs for Negative Control Outcomes in the 2019–2020 Influenza Season (September 30, 2019 – March 7, 2020) for Patients Aged 65+ 0, ≥1, 1–2, and ≥3 Risk Factors

Table S20. Weighted and Adjusted rVEs for Negative Control Outcomes in the 2019–2020 Influenza Season (September 30, 2019 – March 7, 2020) for Patients Aged 65+ 0, ≥1, 1–2, and ≥3 Risk Factors

Figure S1. Week of Any IRME by Vaccine for (a) 0 Risk Factors, (b) ≥1 Risk Factors, (c) 1–2 Risk Factors, and (d) ≥3 Risk Factors

Figure S2. Week of outpatient IRME by vaccine for (a) 0 risk factors, (b) ≥1 Risk Factors, (c) 1–2 Risk Factors, and (d) ≥3 Risk Factors

Figure S3. Week of influenza- or pneumonia-related hospitalization by vaccine for (a) 0 risk factors, (b) ≥1 Risk Factors, (c) 1–2 Risk Factors, and (d) ≥3 Risk Factors

Table S1. Code Sets for Influenza Vaccines, Outcome Measures, and Risk Factors

Category	Variable	Code Type	Codes
Vaccines	aTIV	CVX	168, 205
		CPT	90653, 90689, 90694
		NDC	70461-0019-03, 70461-0019-04
	HD-TIV	CVX	135, 197
		CPT	90662
		NDC	49281-0405-65, 49281-0405-88
Outcomes	Influenza-related medical encounter	ICD-10-CM	J09*, Influenza due to identified novel influenza A viruses<br>J10*, Influenza due to other identified influenza virus<br>J11*, Influenza due to unidentified influenza virus
	Influenza- or pneumonia-related hospitalizations	ICD-10-CM	J09*, Influenza due to identified novel influenza A viruses<br>J10*, Influenza due to other identified influenza virus<br>J11*, Influenza due to unidentified influenza virus<br>J12*, Viral pneumonia, not elsewhere classified<br>J13*, Pneumonia due to Streptococcus pneumoniae<br>J14*, Pneumonia due to Hemophilus influenzae<br>J15*, Bacterial pneumonia, not elsewhere classified<br>J16*, Pneumonia due to other infectious organisms, not elsewhere classified<br>J17*, Pneumonia in diseases classified elsewhere<br>J18*, Pneumonia, unspecified organism
Risk Factors	Asthma	ICD-10-CM	J45*
	Neurologic or neurodevelopmental condition	ICD-10-CM	F01*–F03*, F1027, F1097, F1327, F1917, F1927, F1997, F80*–F89, G10, G20, G21*, G30*, G31*, G35, G40*, G71*–G73*, G80*, G91*, Q03*, Q05*, Q0702, Q0703, Q9351, Q9359, Q937–Q939, Q95*–Q99*, S14*, S24*, S34*
	Blood disorder	ICD-10-CM	D50*–D77

	Chronic lung disease	ICD-10-CM	E84*, J41*–J44*, J47*, J60*–J67*, J684, J701, J703, J811, J8281, J84*
	Endocrine disorder	ICD-10-CM	E01–E13*, E20*–E35*
	Heart disease	ICD-10-CM	I00*–I16*, I20*–I5A, I65*–I68*, I698*, I699*, I70*–I72*, I731, I738*, I739, I74*–I83*, I86*–I99*, K551, K559, K559, Q20*–Q28*, Z958*, Z959
	Renal disease	ICD-10-CM	I12*, I13*, N03*, N05*, N18*, N19, N250, Z49*, Z940, Z992
	Liver disorder	ICD-10-CM	B18*, I85*, I864, K70*, K711*, K713–K715*, K717, K721*, K719*, K73*, K74*, K760, K762–K769, Z944
	Metabolic disorder	ICD-10-CM	E702*–E709, E71*–E83*, E85*–E88*
	Obese with a body mass index of 40 or higher	ICD-10-CM	Z684*
	Weakened immune system	CPT	32851, 32852, 32853, 32854, 33935, 33945, 38240, 38242, 38243, 44135, 44136, 47135, 47136, 48554, 50360, 50365, 50370, 3490F, 80158, 80197, 80169, 80180, 80195
		DRG	001, 002, 005, 006, 007, 008, 019, 650 , 651, 652
		HCPCS	S2053, S2054, S2060, S2152, A4653, A4671, A4672, A4673, A4674, A4680, A4690, A4700, A4705, A4706, A4707, A4708, A4709, A4712, A4714, A4719, A4720, A4721, A4722, A4723, A4724, A4725, A4726, A4728, A4730, A4735, A4736, A4737, A4740, A4750, A4755, A4760, A4765, A4766, A4802, A4820, A4850, A4860, A4870, A4880, A4890, A4900, A4901, A4905, A4910, A4911, A4912, A4913, A4914, A4918, E1500, E1510, E1520, E1530, E1540, E1550, E1560, E1570, E1575, E1580, E1590, E1592, E1594, E1600, E1610, E1615, E1620, E1625, E1629, E1630, E1632, E1634, E1635, E1636, S2065, S2142, S2150, C9006, C9020, C9026, C9106, C9110, C9211, C9212, C9230, C9236, C9239, C9249, C9261, C9421, C9455, J0129, J0135, J0202, J0480, J0490, J0638, J1300, J1602, J1628, J2323, J2793, J3245, J3357, J7504, J7507, J7508, J7513, J7515, J7517, J7520, J8530, J9092, J9094, J9095, J9250, J9260, J9310, J9312, J9330, K0120, K0121, K0412, Q2019, Q2044, Q4079, Q5109, S0087, S0162, S0193, S9359, C9126, C9219, C9264, C9286, C9419, C9420, C9436, C9438, J0215, J0485, J0717, J0718, J1438, J1745, J2350, J2860, J3262, J3358, J3380, J7500, J7501, J7502, J7503, J7505, J7511, J7518, J7525, J7527, J8561,

			J8610, J9010, J9065, J9070, J9080, J9090, J9091, J9093, J9096, J9097, J9311, K0119, K0122, K0123, Q5103, Q5104
		ICD-10-CM	D8481, D811, T8600, T8609, Z48290, Z9481, C00*–C43*, C45*–C76*, C81*–C85*, C88*–C96*, D45, D4622, D471, D474, D479, D47Z1, D47Z9, D6182, D7581, D82*, R75, Z21, D84821, D849, Z482*, Z940, Z941, Z942, Z943, Z944, Z9482, Z9483, T8610, T8619, T8620, T86298, T8630, T8639, T8640, T8649, T86818, T86819, T86858, T86859, T86898, T86899
		ICD-10-PCS	30230AZ, 30230G1, 30230G2, 30230G3, 30230G4, 30230X1, 30230X2, 30230X3, 30230X4, 30230Y1, 30230Y2, 30230Y3, 30230Y4, 30233AZ, 30233G1, 30233G2, 30233G3, 30233G4, 30233X1, 30233X2, 30233X3, 30233X4, 30233Y1, 30233Y2, 30233Y3, 30233Y4, 30240AZ, 30240G1, 30240G2, 30240G3, 30240G4, 30240X1, 30240X2, 30240X3, 30240X4, 30240Y1, 30240Y2, 30240Y3, 30240Y4, 30243AZ, 30243G1, 30243G2, 30243G3, 30243G4, 30243X1, 30243X2, 30243X3, 30243X4, 30243Y1, 30243Y2, 30243Y3, 30243Y4, 30250G1, 30250X1, 30250Y1, 30253G1, 30253X1, 30253Y1, 30260G1, 30260X1, 30260Y1, 30263G1, 30263X1, 30263Y1, 02YA0Z0, 02YA0Z1, 02YA0Z2, 07YM0Z0, 07YM0Z1, 07YM0Z2, 07YP0Z0, 07YP0Z1, 07YP0Z2, 0BYC0Z0, 0BYC0Z1, 0BYC0Z2, 0BYD0Z0, 0BYD0Z1, 0BYD0Z2, 0BYF0Z0, 0BYF0Z1, 0BYF0Z2, 0BYG0Z0, 0BYG0Z1, 0BYG0Z2, 0BYH0Z0, 0BYH0Z1, 0BYH0Z2, 0BYJ0Z0, 0BYJ0Z1, 0BYJ0Z2, 0BYK0Z0, 0BYK0Z1, 0BYK0Z2, 0BYL0Z0, 0BYL0Z1, 0BYL0Z2, 0BYM0Z0, 0BYM0Z1, 0BYM0Z2, 0DY50Z0, 0DY50Z1, 0DY50Z2, 0DY60Z0, 0DY60Z1, 0DY60Z2, 0DY80Z0, 0DY80Z1, 0DY80Z2, 0DYE0Z0, 0DYE0Z1, 0DYE0Z2, 0FY00Z0, 0FY00Z1, 0FY00Z2, 0FYG0Z0, 0FYG0Z1, 0FYG0Z2, 0TY00Z0, 0TY00Z1, 0TY00Z2, 0TY10Z0, 0TY10Z1, 0TY10Z2, 3E1M39Z, 5A1D00Z, 5A1D70Z, 5A1D80Z, 5A1D90Z, BT2900Z, BT290ZZ, BT2910Z, BT291ZZ, BT29Y0Z, BT29YZZ, BT29ZZZ, BT39Y0Z, BT39YZZ, BT39ZZZ, BT49ZZZ
		Medications	abatacept, adalimumab, alefacept, alemtuzumab, anakinra, apremilast, azacitidine, azathioprine, baricitinib, basiliximab, belatacept, belimumab, bendamustine hcl, brodalumab, busulfan, canakinumab, capecitabine,

			carmustine, certolizumab , chlorambucil, cladribine, clofarabine, cyclophosphamide, cyclosporine, cytarabine, dacarbazine, daclizumab, decitabine, dimethyl fumarate, diroximel fumarate, eculizumab, efalizumab, emapalumab, etanercept, everolimus, fingolimod hcl, floxuridine, fludarabine phosphate, fluorouracil, gemcitabine hcl, golimumab, guselkumab, ifosfamide, ifosfamide/mesna, inebilizumab, infliximab, ixekizumab, leflunomide, lenalidomide, lomustine, melphalan, mercaptopurine, methotrexate, muromonab, mycophenolate mofetil, natalizumab, nelarabine, ocrelizumab, ofatumumab, ozanimod hydrochloride, pemetrexed disodium, pirfenidone, pomalidomide, pralatrexate, ravulizumab, rilonacept, risankizumab, sarilumab, satralizumab, secukinumab, siltuximab, siponimod, sirolimus, streptozocin, tacrolimus, temozolomide, temsirolimus, teprotumumab, teriflunomide, thalidomide, thiotepa, tildrakizumab, tocilizumab, tofacitinib citrate, upadacitinib, ustekinumab, vedolizumab
	Had a stroke	ICD-10-CM	G436*, G450–G452, G458, G459, G460–G462, I60*–I63*, I690*–I693*, Z8673

aTIV, adjuvanted trivalent inactivated influenza vaccine; CPT, Current Procedural Terminology; CVX, vaccine codes; HD-TIV, high-dose trivalent inactivated influenza vaccine; ICD-10-CM, International Classification of Diseases – Ttenth edition – Clinical Modification; NDC, national drug codes

Table S2. Covariate Definitions

Variable	Description	Variable Type
**Age**	Age in years at the Index Date	Continuous (years of age at index date); Categorical: 65–74, 75–84, ≥85
**Sex**	Recorded Sex in Pre-Index period or Index Date	Categorical: Female, Male
**Race**	Recorded Race in Pre-Index period	Categorical: Asian, Black, White, Other, Not Reported
**Ethnicity**	Recorded Ethnicity in Pre-Index period	Categorical: Hispanic, Non-Hispanic, Not Reported
**Geographic Region**	Geographic Region of residence recorded at index date	Patient home location based on zip-3 postal codes and categorized into one of four mutually exclusive geographic locations in the U.S. based on regional census divisions: Northeast, Midwest, South, West.
**Week of Vaccination**	Week of vaccination with aTIV or HD-TIV (number of weeks since the start of the Vaccination Identification Period)	Integer value indicating the number of weeks since the start of the Vaccination Identification Period, beginning with 1, during which the patient was vaccinated<br>Also shown by calendar month in descriptive tables as categorical: August, September, October, November, December, January
**Frailty Index**	Electronic frailty index score calculated based on records in the pre-index period	Continuous; defined according to Faurot et al.[a]
**Clinical Categories of Risk Factors**	Asthma	Dichotomous: Yes, No
	Neurologic or neurodevelopmental condition	Dichotomous: Yes, No
	Blood disorder	Dichotomous: Yes, No
	Chronic lung disease	Dichotomous: Yes, No
	Endocrine disorder	Dichotomous: Yes, No
	Heart disease	Dichotomous: Yes, No
	Renal disease	Dichotomous: Yes, No
	Liver disorder	Dichotomous: Yes, No
	Metabolic disorder	Dichotomous: Yes, No
	Obese with a body mass index of 40 or higher	Dichotomous: Yes, No
	Weakened immune system	Dichotomous: Yes, No
	Had a stroke	Dichotomous: Yes, No

Variable	Description	Variable Type
**Healthcare Resource Utilization**	Number of outpatient visits in the one year prior to vaccination date (Pre-index period).	Count, from claims
	Number of all-cause hospital admissions in the pre-index period	Count, from claims
	Number of all-cause emergency department visits in the pre-index period	Count, from claims

aTIV: adjuvanted trivalent inactivated influenza vaccine; HD-TIV high-dose trivalent inactivated influenza vaccine;

[a] Faurot KR, Jonsson Funk M, Pate V, et al. Using claims data to predict dependency in activities of daily living as a proxy for frailty. *Pharmacoepidemiol Drug Saf*. 2015;24(1):59-66.

Table S3. Demographic Characteristics and SMDs Before and After IPTW in the 2019–2020 Influenza Season (September 29, 2019 – March 7, 2020) by Vaccination Cohort Among Patients with 0 Risk Factors

	**0 Risk Factors**					
	**aTIV**		**HD-TIV**		**Unweighted SMD**	**IPTW SMD**
	N=161,018		N=308,048			
	N/Mean	%/SD	N/Mean	%/SD		
**Age, Index Date (Mean, SD)**	73.2	6.0	73.1	6.1	0.01	0.01
**Age Group, Index Date (N, %)**						
65-74	102,267	63.5%	196,984	63.9%	0.01	0.01
75-84	46,195	28.7%	85,909	27.9%	0.02	0.00
85+	12,556	7.8%	25,155	8.2%	0.01	0.01
**Sex (N, %)**						
Female	97,626	60.6%	186,091	60.4%	0.00	0.00
Male	63,392	39.4%	121,957	39.6%	0.00	0.00
**Race (N, %)**						
White	85,676	53.2%	160,155	52.0%	0.02	0.00
Black	2,881	1.8%	5,894	1.9%	0.01	0.00
Asian	1,914	1.2%	4,642	1.5%	0.03	0.00
Other	1,291	0.8%	2,621	0.9%	0.01	0.00
Unknown/Not Reported	69,256	43.0%	134,736	43.7%	0.01	0.00
**Ethnicity (N, %)**						
Hispanic	2,840	1.8%	6,009	2.0%	0.01	0.00
Non-Hispanic	127,528	79.2%	243,476	79.0%	0.00	0.00
Unknown/Not Reported	30,650	19.0%	58,563	19.0%	0.00	0.00
**Geographic Region (N, %)**						
Northeast	21,873	13.6%	42,629	13.8%	0.01	0.01
Midwest	23,879	14.8%	74,874	24.3%	**0.24**	0.01
South	97,439	60.5%	115,757	37.6%	**0.47**	0.00
West	17,827	11.1%	74,788	24.3%	**0.35**	0.02

aTIV: adjuvanted trivalent inactivated influenza vaccine; HD-TIV high-dose trivalent inactivated influenza vaccine; IPTW: inverse probability of treatment weighting; SD: standard deviation; SMD: standardized mean difference

Table S4. Demographic Characteristics and SMDs Before and After IPTW in the 2019–2020 Influenza Season (September 29, 2019 – March 7, 2020) by Vaccination Cohort Among Patients with ≥1 Risk Factors

	≥1 Risk Factors					
	aTIV		HD-TIV		Unweighted SMD	IPTW SMD
	N= 954,707		N=2,253,670			
	N/Mean	%/SD	N/Mean	%/SD		
**Age, Index Date (Mean, SD)**	74.9	6.2	74.9	6.3	0.01	0.00
**Age Group, Index Date (N, %)**						
65-74	492,588	51.6%	1,170,472	51.9%	0.01	0.00
75-84	342,230	35.8%	787,636	34.9%	0.02	0.00
85+	119,889	12.6%	295,562	13.1%	0.02	0.01
**Sex (N, %)**						
Female	550,456	57.7%	1,305,406	57.9%	0.01	0.00
Male	404,251	42.3%	948,264	42.1%	0.01	0.00
**Race (N, %)**						
White	497,822	52.1%	1,172,086	52.0%	0.00	0.00
Black	32,069	3.4%	90,619	4.0%	0.04	0.00
Asian	12,124	1.3%	39,727	1.8%	0.04	0.00
Other	9,159	1.0%	24,191	1.1%	0.01	0.00
Unknown/Not Reported	403,533	42.3%	927,047	41.1%	0.02	0.00
**Ethnicity (N, %)**						
Hispanic	25,463	3%	65,645	2.9%	0.01	0.00
Non-Hispanic	787,076	82%	1,854,030	82.3%	0.00	0.00
Unknown/Not Reported	142,168	15%	333,995	14.8%	0.00	0.00
**Geographic Region (N, %)**						
Northeast	152,961	16.0%	446,445	19.8%	0.10	0.01
Midwest	166,015	17.4%	495,821	22.0%	**0.12**	0.00
South	517,531	54.2%	851,753	37.8%	**0.33**	0.00
West	118,200	12.4%	459,651	20.4%	**0.22**	0.02

aTIV: adjuvanted trivalent inactivated influenza vaccine; HD-TIV high-dose trivalent inactivated influenza vaccine; IPTW: inverse probability of treatment weighting; SD: standard deviation; SMD: standardized mean difference

Table S5. Demographic Characteristics and SMDs Before and After IPTW in the 2019–2020 Influenza Season (September 29, 2019 – March 7, 2020) by Vaccination Cohort Among Patients 1–2 Risk Factors

	**1–2 Risk Factors**					
	**aTIV**		**HD-TIV**		**Unweighted SMD**	**IPTW SMD**
	N=356,743		N=794,986			
	N/Mean	%/SD	N/Mean	%/SD		
**Age, Index Date (Mean, SD)**	74.0	6.1	73.9	6.2	0.02	0.00
**Age Group, Index Date (N, %)**						
65-74	207,552	58.2%	468,420	58.9%	0.02	0.00
75-84	113,992	32.0%	245,912	30.9%	0.02	0.00
85+	35,199	9.9%	80,654	10.1%	0.01	0.01
**Sex (N, %)**						
Female	209,615	58.8%	467,217	58.8%	0.00	0.00
Male	147,128	41.2%	327,769	41.2%	0.00	0.00
**Race (N, %)**						
White	193,682	54.3%	431,178	54.2%	0.00	0.00
Black	8,655	2.4%	22,026	2.8%	0.02	0.00
Asian	4,292	1.2%	13,225	1.7%	0.04	0.00
Other	2,942	0.8%	7,425	0.9%	0.01	0.00
Unknown/Not Reported	147,172	41.3%	321,132	40.4%	0.02	0.00
**Ethnicity (N, %)**						
Hispanic	7,427	2.1%	18,430	2.3%	0.02	0.00
Non-Hispanic	290,138	81.3%	646,888	81.4%	0.00	0.00
Unknown/Not Reported	59,178	16.6%	129,668	16.3%	0.01	0.00
**Geographic Region (N, %)**						
Northeast	56,419	15.8%	151,047	19.0%	0.08	0.01
Midwest	61,637	17.3%	181,555	22.8%	**0.14**	0.01
South	194,100	54.4%	290,355	36.5%	**0.37**	0.00
West	44,587	12.5%	172,029	21.6%	**0.24**	0.02

aTIV: adjuvanted trivalent inactivated influenza vaccine; HD-TIV high-dose trivalent inactivated influenza vaccine; IPTW: inverse probability of treatment weighting; SD: standard deviation; SMD: standardized mean difference

Table S6. Demographic Characteristics and SMDs Before and After IPTW in the 2019–2020 Influenza Season (September 29, 2019 – March 7, 2020) by Vaccination Cohort Among Patients with ≥3 Risk Factors

	≥3 Risk Factors					
	**aTIV**		**HD-TIV**		**Unweighted SMD**	**IPTW SMD**
	N=597,964		N=1,458,684			
	N/Mean	%/SD	N/Mean	%/SD		
**Age, Index Date (Mean, SD)**	75.5	6.3	75.4	6.4	0.01	0.00
**Age Group, Index Date (N, %)**						
65-74	285,036	47.7%	702,052	48.1%	0.01	0.00
75-84	228,238	38.2%	541,724	37.1%	0.02	0.00
85+	84,690	14.2%	214,908	14.7%	0.02	0.01
**Sex (N, %)**						
Female	340,841	57.0%	838,189	57.5%	0.01	0.00
Male	257,123	43.0%	620,495	42.5%	0.01	0.00
**Race (N, %)**						
White	304,140	50.9%	740,908	50.8%	0.00	0.00
Black	23,414	3.9%	68,593	4.7%	0.04	0.00
Asian	7,832	1.3%	26,502	1.8%	0.04	0.00
Other	6,217	1.0%	16,766	1.1%	0.01	0.00
Unknown/Not Reported	256,361	42.9%	605,915	41.5%	0.03	0.00
**Ethnicity (N, %)**						
Hispanic	18,036	3.0%	47,215	3.2%	0.01	0.00
Non-Hispanic	496,938	83.1%	1,207,142	82.8%	0.01	0.00
Unknown/Not Reported	82,990	13.9%	204,327	14.0%	0.00	0.00
**Geographic Region (N, %)**						
Northeast	96,542	16.1%	295,398	20.3%	**0.11**	0.01
Midwest	104,378	17.5%	314,266	21.5%	0.10	0.00
South	323,431	54.1%	561,398	38.5%	**0.32**	0.00
West	73,613	12.3%	287,622	19.7%	**0.20**	0.01

aTIV: adjuvanted trivalent inactivated influenza vaccine; HD-TIV high-dose trivalent inactivated influenza vaccine; IPTW: inverse probability of treatment weighting; SD: standard deviation; SMD: standardized mean difference

Table S7. Clinical Characteristics and SMDs Before and After IPTW in the 2019–2020 Influenza Season (September 29, 2019 – March 7, 2020) by Vaccination Cohort Among Patients with 0 Risk Factors

	**0 Risk Factors**					
	**aTIV**		**HD-TIV**		**Unweighted SMD**	**IPTW SMD**
	N=161,018		N=308,048			
	N/Mean /Median	%/SD /IQR	N/Mean /Median	%/SD /IQR		
**Month of Vaccination**						
August	9,412	5.8%	3,463	1.1%	**0.26**	**0.19**
September	42,625	26.5%	75,720	24.6%	0.04	0.10
October	74,858	46.5%	139,009	45.1%	0.03	0.00
November	23,682	14.7%	57,479	18.7%	**0.11**	0.00
December	7,439	4.6%	21,610	7.0%	0.10	0.03
January	3,002	1.9%	10,767	3.5%	0.10	0.05
**Risk Factors, N (%)**						
Heart Disease						
Metabolic disorders						
Endocrine disorders						
Blood disorders						
Weakened immune system						
Kidney diseases						
Chronic lung disease						
Asthma						
Stroke						
Neurologic and neurodevelopmental conditions						
Liver disorders						
Obesity (body mass index ≥40)						
**Baseline All-cause Healthcare Utilization**						
Number of OP visits (Mean, SD)	1.5	2.4	1.6	2.4	0.02	0.00
Number of OP visits (Median, IQR)	1	2	1	2		
Number of IP admissions (Mean, SD)	0.0	0.3	0.0	0.3	0.00	0.00
Number of IP admissions (Median, IQR)	0	0	0	0		
Number of ER visits (Mean, SD)	0.1	0.3	0.1	0.3	0.01	0.00
Number of ER visits (Median, IQR)	0	0	0	0		
**Electronic Frailty Index Score (Mean, SD)**	7.8%	4.4%	7.8%	4.5%	0.00	0.00
Electronic Frailty Index Score (Median, IQR)	7.5%	4.2%	7.4%	4.3%		
**Electronic Frailty Index Score (N, %)**						
<5%	37,748	23.4%	73,644	23.9%	0.01	0.00
≥5% to < 20%	120,470	74.8%	229,015	74.3%	0.01	0.00
≥20%	2,800	1.7%	5,389	1.7%	0.00	0.00

aTIV: adjuvanted trivalent inactivated influenza vaccine; ER, emergency room; HD-TIV high-dose trivalent inactivated influenza vaccine; IP: inpatient; IPTW: inverse probability of treatment weighting; IQR: interquartile range; OP: outpatient; SD: standard deviation; SMD: standardized mean difference

Table S8. Clinical Characteristics and SMDs Before and After IPTW in the 2019–2020 Influenza Season (September 29, 2019 – March 7, 2020) by Vaccination Cohort Among Patients with ≥1 Risk Factors

	≥1 Risk Factors					
	**aTIV**		**HD-TIV**		**Unweighted SMD**	**IPTW SMD**
	N= 954,707		N=2,253,670			
	N/Mean /Median	%/SD /IQR	N/Mean /Median	%/SD /IQR		
**Month of Vaccination**						
August	63,839	6.7%	24,673	1.1%	**0.29**	**0.21**
September	264,513	27.7%	572,910	25.4%	0.05	0.10
October	433,038	45.4%	1,036,147	46.0%	0.01	0.03
November	134,560	14.1%	400,883	17.8%	0.10	0.01
December	41,939	4.4%	150,393	6.7%	0.10	0.03
January	16,818	1.8%	68,664	3.0%	0.08	0.06
**Risk Factors, N (%)**						
Heart Disease	798,053	83.6%	1,910,177	84.8%	0.03	0.00
Metabolic disorders	700,335	73.4%	1,669,957	74.1%	0.02	0.00
Endocrine disorders	490,125	51.3%	1,184,655	52.6%	0.02	0.00
Blood disorders	237,501	24.9%	579,563	25.7%	0.02	0.00
Weakened immune system	180,722	18.9%	430,562	19.1%	0.00	0.00
Kidney diseases	168,361	17.6%	435,678	19.3%	0.04	0.00
Chronic lung disease	148,701	15.6%	373,770	16.6%	0.03	0.00
Asthma	80,064	8.4%	200,147	8.9%	0.02	0.00
Stroke	70,794	7.4%	184,652	8.2%	0.03	0.00
Neurologic and neurodevelopmental conditions	61,908	6.5%	165,989	7.4%	0.03	0.00
Liver disorders	48,505	5.1%	118,459	5.3%	0.01	0.00
Obesity (body mass index ≥40)	40,398	4.2%	111,160	4.9%	0.03	0.00
**Baseline All-cause Healthcare Utilization**						
Number of OP visits (Mean, SD)	6.6	6.0	6.7	6.1	0.01	0.00
Number of OP visits (Median, IQR)	5	7	5	7		
Number of IP admissions (Mean, SD)	0.2	1.0	0.3	1.0	0.02	0.00
Number of IP admissions (Median, IQR)	0	0	0	0		
Number of ER visits (Mean, SD)	0.4	1.0	0.4	1.0	0.03	0.00
Number of ER visits (Median, IQR)	0	0	0	0		
**Electronic Frailty Index Score (Mean, SD)**	12.3%	14.4%	13.2%	15.7%	0.06	0.06
Electronic Frailty Index Score (Median, IQR)	7.6%	8.9%	7.7%	9.7%		
**Electronic Frailty Index Score (N, %)**						
<5%	278,001	29.1%	641,191	28.5%	0.01	0.00
≥5% to < 20%	535,661	56.1%	1,241,572	55.1%	0.02	0.00
≥20%	141,045	14.8%	370,907	16.5%	0.05	0.00

aTIV: adjuvanted trivalent inactivated influenza vaccine; ER, emergency room; HD-TIV high-dose trivalent inactivated influenza vaccine; IP: inpatient; IPTW: inverse probability of treatment weighting; IQR: interquartile range; OP: outpatient; SD: standard deviation; SMD: standardized mean difference

Table S9. Clinical Characteristics and SMDs Before and After IPTW in the 2019–2020 Influenza Season (September 29, 2019 – March 7, 2020) by Vaccination Cohort Among Patients with 1–2 Risk Factors

	1–2 Risk Factors					
	**aTIV**		**HD-TIV**		**Unweighted SMD**	**IPTW SMD**
	N=356,743		N=794,986			
	N/Mean /Median	%/SD /IQR	N/Mean /Median	%/SD /IQR		
**Month of Vaccination**						
August	21,934	6.1%	8,567	1.1%	**0.27**	**0.20**
September	95,751	26.8%	195,043	24.5%	0.05	0.10
October	165,098	46.3%	366,237	46.1%	0.00	0.02
November	51,735	14.5%	144,685	18.2%	0.10	0.01
December	15,799	4.4%	54,763	6.9%	**0.11**	0.03
January	6,426	1.8%	25,691	3.2%	0.09	0.05
**Risk Factors, N (%)**						
Heart Disease	225,708	63.3%	509,730	64.1%	0.02	0.00
Metabolic disorders	168,191	47.1%	378,470	47.6%	0.01	0.00
Endocrine disorders	74,934	21.0%	167,425	21.1%	0.00	0.00
Blood disorders	17,550	4.9%	37,204	4.7%	0.01	0.00
Weakened immune system	33,029	9.3%	70,653	8.9%	0.01	0.00
Kidney diseases	6,921	1.9%	15,998	2.0%	0.01	0.00
Chronic lung disease	14,431	4.0%	33,912	4.3%	0.01	0.00
Asthma	9,596	2.7%	22,041	2.8%	0.01	0.00
Stroke	3,867	1.1%	8,483	1.1%	0.00	0.00
Neurologic and neurodevelopmental conditions	7,695	2.2%	18,694	2.4%	0.01	0.00
Liver disorders	4,693	1.3%	9,898	1.2%	0.01	0.00
Obesity (body mass index ≥40)	3,089	0.9%	8,170	1.0%	0.02	0.00
**Baseline All-cause Healthcare Utilization**						
Number of OP visits (Mean, SD)	4.2	4.1	4.3	4.1	0.02	0.00
Number of OP visits (Median, IQR)	3	5	3	5		
Number of IP admissions (Mean, SD)	0.1	0.6	0.1	0.5	0.01	0.00
Number of IP admissions (Median, IQR)	0	0	0	0		
Number of ER visits (Mean, SD)	0.2	0.6	0.2	0.6	0.01	0.00
Number of ER visits (Median, IQR)	0	0	0	0		
**Electronic Frailty Index Score (Mean, SD)**	8.9%	8.3%	8.9%	8.5%	0.01	0.01
Electronic Frailty Index Score (Median, IQR)	6.6%	6.2%	6.6%	6.3%		
**Electronic Frailty Index Score (N, %)**						
<5%	117,768	33.0%	263,427	33.1%	0.00	0.00
≥5% to < 20%	214,714	60.2%	475,221	59.8%	0.01	0.00
≥20%	24,261	6.8%	56,338	7.1%	0.01	0.00

aTIV: adjuvanted trivalent inactivated influenza vaccine; ER, emergency room; HD-TIV high-dose trivalent inactivated influenza vaccine; IP: inpatient; IPTW: inverse probability of treatment weighting; IQR: interquartile range; OP: outpatient; SD: standard deviation; SMD: standardized mean difference

Table S10. Clinical Characteristics and SMDs Before and After IPTW in the 2019–2020 Influenza Season (September 29, 2019 – March 7, 2020) by Vaccination Cohort Among Patients with ≥3 Risk Factors

	≥3 Risk Factors					
	**aTIV**		**HD-TIV**		**Unweighted SMD**	**IPTW SMD**
	N=597,964		N=1,458,684			
	N/Mean /Median	%/SD /IQR	N/Mean /Median	%/SD /IQR		
**Month of Vaccination**						
August	41,905	7.0%	16,106	1.1%	**0.30**	**0.22**
September	168,762	28.2%	377,867	25.9%	0.05	0.10
October	267,940	44.8%	669,910	45.9%	0.02	0.04
November	82,825	13.9%	256,198	17.6%	0.10	0.01
December	26,140	4.4%	95,630	6.6%	0.10	0.04
January	10,392	1.7%	42,973	2.9%	0.08	0.07
**Risk Factors, N (%)**						
Heart Disease	572,345	95.7%	1,400,447	96.0%	0.01	0.00
Metabolic disorders	532,144	89.0%	1,291,487	88.5%	0.01	0.00
Endocrine disorders	415,191	69.4%	1,017,230	69.7%	0.01	0.00
Blood disorders	219,951	36.8%	542,359	37.2%	0.01	0.00
Weakened immune system	147,693	24.7%	359,909	24.7%	0.00	0.00
Kidney diseases	161,440	27.0%	419,680	28.8%	0.04	0.00
Chronic lung disease	134,270	22.5%	339,858	23.3%	0.02	0.00
Asthma	70,468	11.8%	178,106	12.2%	0.01	0.00
Stroke	66,927	11.2%	176,169	12.1%	0.03	0.00
Neurologic and neurodevelopmental conditions	54,213	9.1%	147,295	10.1%	0.04	0.00
Liver disorders	43,812	7.3%	108,561	7.4%	0.00	0.00
Obesity (body mass index ≥40)	37,309	6.2%	102,990	7.1%	0.03	0.00
**Baseline All-cause Healthcare Utilization**						
Number of OP visits (Mean, SD)	8.0	6.5	7.9	6.5	0.01	0.01
Number of OP visits (Median, IQR)	7	8	6	8		
Number of IP admissions (Mean, SD)	0.3	1.2	0.4	1.1	0.02	0.00
Number of IP admissions (Median, IQR)	0	0	0	0		
Number of ER visits (Mean, SD)	0.5	1.1	0.5	1.2	0.03	0.01
Number of ER visits (Median, IQR)	0	1	0	1		
**Electronic Frailty Index Score (Mean, SD)**	14.4%	16.7%	15.4%	18.1%	0.06	0.06
Electronic Frailty Index Score (Median, IQR)	8.2%	11.3%	8.5%	12.5%		
**Electronic Frailty Index Score (N, %)**						
<5%	160,233	26.8%	377,764	25.9%	0.02	0.00
≥5% to < 20%	320,947	53.7%	766,351	52.5%	0.02	0.00
≥20%	116,784	19.5%	314,569	21.6%	0.05	0.00

aTIV: adjuvanted trivalent inactivated influenza vaccine; ER, emergency room; HD-TIV high-dose trivalent inactivated influenza vaccine; IP: inpatient; IPTW: inverse probability of treatment weighting; IQR: interquartile range; OP: outpatient; SD: standard deviation; SMD: standardized mean difference

Table S11. Sample Size Pre- and Post-weighting

	0 Risk Factors		≥1 Risk Factors		≥3 Risk Factors		1-2 Risk Factors	
	aTIV	HD-TIV	aTIV	HD-TIV	aTIV	HD-TIV	aTIV	HD-TIV
Pre-weighting	161,018	308,048	954,707	2,253,670	597,964	1,458,684	356,743	794,986
Post-weighting	161,998	307,779	961,148	2,251,446	602,213	1,457,177	358,855	794,308

aTIV: adjuvanted trivalent inactivated influenza vaccine; HD-TIV high-dose trivalent inactivated influenza vaccine

Table S12. Most Common Factors Among Patients with 1 Risk Factor

1 Risk Factor		
N = 453,076		
**Most Common Factor**	**Patient Count**	**%**
Heart disease	188,767	41.7%
Metabolic disorders	98,676	21.8%
Endocrine disorders	69,271	15.3%
Weakened immune system	41,322	9.1%
Chronic lung disease	13,346	2.9%
Blood disorders	11,934	2.6%
Asthma	9,370	2.1%
Neurologic and neurodevelopmental conditions	8,147	1.8%
Liver disorders	4,215	0.9%
Kidney diseases	3,077	0.7%

Table S13. Most Common Conditions Among Patients with 2 Risk Factors

2 Risk Factors			
N = 698,653			
**Most Common Condition 1**	**Most Common Condition 2**	**Patient Count**	**%**
Heart disease	Metabolic disorders	340,555	48.7%
Heart disease	Endocrine disorders	87,287	12.5%
Endocrine disorders	Metabolic disorders	59,819	8.6%
Heart disease	Weakened immune system	29,144	4.2%
Chronic lung disease	Heart disease	20,365	2.9%
Blood disorders	Heart disease	19,682	2.8%
Metabolic disorders	Weakened immune system	14,315	2.0%
Heart disease	Kidney diseases	12,861	1.8%
Blood disorders	Heart disease	11,057	1.6%
Metabolic disorders	Weakened immune system	10,612	1.5%

Table S14. Most Common Conditions Among Patients with 3 Risk Factors

**3 Risk Factors**				
**N = 800,732**				
**Most Common Condition 1**	**Most Common Condition 2**	**Most Common Condition 3**	**Patient Count**	**%**
Endocrine disorders	Heart disease	Metabolic disorders	335,538	41.9%
Blood disorders	Heart disease	Metabolic disorders	60,270	7.5%
Heart disease	Metabolic disorders	Weakened immune system	57,081	7.1%
Heart disease	Metabolic disorders	Kidney diseases	40,770	5.1%
Heart disease	Metabolic disorders	Chronic lung disease	39,914	5.0%
Heart disease	Metabolic disorders	Stroke	20,937	2.6%
Asthma	Heart disease	Metabolic disorders	20,910	2.6%
Heart disease	Metabolic disorders	Neurologic and neurodevelopmental conditions	16,576	2.1%
Endocrine disorders	Heart disease	Weakened immune system	14,493	1.8%
Blood disorders	Endocrine disorders	Heart disease	14,155	1.8%

Table S15. Unweighted Exposure-Outcome Results Table

Outcome	aTIV		HD-TIV	
	N/Mean	%/SD	N/Mean	%/SD
0 Risk Factors	161,018	100.0%	308,048	100.0%
Any IRME	477	0.30%	902	0.29%
Outpatient IRME	438	0.27%	847	0.27%
Influenza- or pneumonia-related hospitalization	232	0.14%	389	0.13%
≥1 Risk Factors	954,707	100.0%	2,253,670	100.0%
Any IRME	6,641	0.70%	17,088	0.76%
Outpatient IRME	5,671	0.59%	14,386	0.64%
Influenza- or pneumonia-related hospitalization	7,235	0.76%	19,608	0.87%
1–2 Risk Factors	356,743	100.0%	794,986	100.0%
Any IRME	1,722	0.48%	4,312	0.54%
Outpatient IRME	1,580	0.44%	3,976	0.50%
Influenza- or pneumonia-related hospitalization	959	0.27%	2,219	0.28%
≥3 Risk Factors	597,964	100.0%	1,458,684	100.0%
Any IRME	4,919	0.82%	12,776	0.88%
Outpatient IRME	4,091	0.68%	10,410	0.71%
Influenza- or pneumonia-related hospitalization	6,276	1.05%	17,389	1.19%

aTIV: adjuvanted trivalent influenza vaccine; HD-TIV: high-dose trivalent influenza vaccine; IRME: influenza-related medical encounter; SD: standard deviation

Table S16. Weighted Exposure-Outcome Results Table

Outcome	aTIV		HD-TIV	
	N/Mean	%/SD	N/Mean	%/SD
0 Risk Factors	161,998	100.0%	307,779	100.0%
Any IRME	473	0.29%	945	0.31%
Outpatient IRME	432	0.27%	888	0.29%
Influenza- or pneumonia-related hospitalization	218	0.13%	397	0.13%
≥1 Risk Factors	961,148	100.0%	2,251,446	100.0%
Any IRME	6,500	0.68%	17,396	0.77%
Outpatient IRME	5,460	0.57%	14,745	0.65%
Influenza- or pneumonia-related hospitalization	7,440	0.77%	19,482	0.87%
1–2 Risk Factors	358,855	100.0%	794,308	100.0%
Any IRME	1,638	0.46%	4,446	0.56%
Outpatient IRME	1,501	0.42%	4,107	0.52%
Influenza- or pneumonia-related hospitalization	927	0.26%	2,255	0.28%
≥3 Risk Factors	602,213	100.0%	1,457,177	100.0%
Any IRME	4,811	0.80%	12,995	0.89%
Outpatient IRME	3,926	0.65%	10,667	0.73%
Influenza- or pneumonia-related hospitalization	6,385	1.06%	17,347	1.19%

aTIV: adjuvanted trivalent influenza vaccine; HD-TIV: high-dose trivalent influenza vaccine; IRME: influenza-related medical encounter; SD: standard deviation

Table S17. Unweighted and Unadjusted rVEs for Any IRME, Outpatient IRME, and Influenza- or Pneumonia-related Hospitalization in the 2019–2020 Influenza Season (September 30, 2019 – March 7, 2020) for Patients Aged 65+

	0 Risk Factors			≥1 Risk Factors			1–2 Risk Factors			≥3 Risk Factors		
	rVE	95% CI		rVE	95% CI		rVE	95% CI		rVE	95% CI	
		UCL	LCL		UCL	LCL		UCL	UCL		UCL	UCL
Any IRME	-1.2	-13.1	9.5	8.3	5.7	10.9	11.1	5.9	15.9	6.1	3.0	9.2
Outpatient IRME	1.1	-11.0	11.9	7.0	4.1	9.8	11.5	6.2	16.5	4.2	0.6	7.6
Influenza- or pneumonia-related hospitalization	-14.1	-34.3	3.0	13.0	10.6	15.3	3.7	-3.9	10.7	12.1	9.5	14.6

CI: confidence limit; IRME: influenza-related medical encounters; LCL: lower confidence limit; rVE: relative vaccine effectiveness; UCL: upper confidence limit

Table S18. Days in Time from Vaccination to Outcome; weighted

Outcome	aTIV			HD-TIV		
	Days, Median	1st quartile	3rd quartile	Days, Median	1st quartile	3rd quartile
0 Risk Factors						
Any IRME	103	75	129	103	76	129
Outpatient IRME	105	76	129	102	75	128
Influenza- or pneumonia-related hospitalization	94	60	126	88	55	121
≥1 Risk Factors						
Any IRME	104	75	131	105	76	130
Outpatient IRME	105	75	131	105	76	129
Influenza- or pneumonia-related hospitalization	85	53	118	86	54	119
1–2 Risk Factors						
Any IRME	103	74	131	104	76	128
Outpatient IRME	103	74	131	104	76	128
Influenza- or pneumonia-related hospitalization	87	53	120	87	56	120
≥3 Risk Factors						
Any IRME	104	75	130	105	77	130
Outpatient IRME	105	76	131	105	76	130
Influenza- or pneumonia-related hospitalization	85	53	118	86	53	119

aTIV: adjuvanted trivalent influenza vaccine; body mass index HD-TIV: high-dose trivalent influenza vaccine; IRME: influenza-related medical encounter

Table S19. Unweighted and Unadjusted rVEs for Negative Control Outcomes in the 2019–2020 Influenza Season (September 30, 2019 – March 7, 2020) for Patients Aged 65+ 0, ≥1, 1–2, and ≥3 Risk Factors

	0 Risk Factors			≥1 Risk Factors			1–2 Risk Factors			≥3 Risk Factors		
	rVE	95% CI		rVE	95% CI		rVE	95% CI		rVE	95% CI	
		LCL	UCL		LCL	UCL		LCL	UCL		LCL	UCL
Appendicitis	-32.1	-90.0	8.2	-5.5	-18.7	6.2	-17.1	-43.3	4.3	-0.6	-16.3	13.1
Cataracts	-13.5	-16.4	-10.8	-5.7	-6.6	-4.9	-8.2	-9.7	-6.7	-4.9	-6.0	-3.8
Eyelid disorders	-17.7	-23.4	-12.3	-9.2	-10.7	-7.6	-12.9	-15.8	-10.1	-8.1	-10.0	-6.3
Ingrown nails	-22.7	-34.8	-11.6	-5.1	-7.2	-3.0	-10.5	-15.3	-5.9	-5.1	-10.3	-0.2
Lipomas	-8.0	-25.0	6.7	-4.3	-8.6	-0.2	-4.0	-11.8	3.1	-0.6	-16.3	13.1

CI: confidence limit; LCL: lower confidence limit; rVE: relative vaccine effectiveness; UCL: upper confidence limit

Table S20. Weighted and Adjusted rVEs for Negative Control Outcomes in the 2019–2020 Influenza Season (September 30, 2019 – March 7, 2020) for Patients Aged 65+ 0, ≥1, 1–2, and ≥3 Risk Factors

	0 Risk Factors			≥1 Risk Factors			1–2 Risk Factors			≥3 Risk Factors		
	rVE	95% CI		rVE	95% CI		rVE	95% CI		rVE	95% CI	
		LCL	UCL		LCL	UCL		LCL	UCL		LCL	UCL
Appendicitis	-40.3	-100.8	1.9	-1.2	-14.0	10.2	-16.0	-42.0	5.2	5.3	-9.8	18.3
Cataracts	-5.4	-8.1	-2.8	-0.5	-1.4	0.3	-2.4	-3.9	-1.0	0.4	-0.7	1.4
Eyelid disorders	-6.7	-12.0	-1.7	-1.7	-3.2	-0.3	-4.6	-7.4	-2.0	-0.5	-2.3	1.3
Ingrown nails	-16.0	-27.9	-5.2	-5.1	-7.3	-3.0	-8.6	-13.3	-4.0	-4.3	-6.7	-1.9
Lipomas	-3.6	-20.0	10.5	1.2	-2.9	5.1	-0.1	-7.6	6.8	1.8	-3.2	6.5

CI: confidence limit; LCL: lower confidence limit; rVE: relative vaccine effectiveness; UCL: upper confidence limit

Figure S1. Week of Any IRME by Vaccine for (a) 0 Risk Factors, (b) ≥1 Risk Factors, (c) 1–2 Risk Factors, and (d) ≥3 Risk Factors

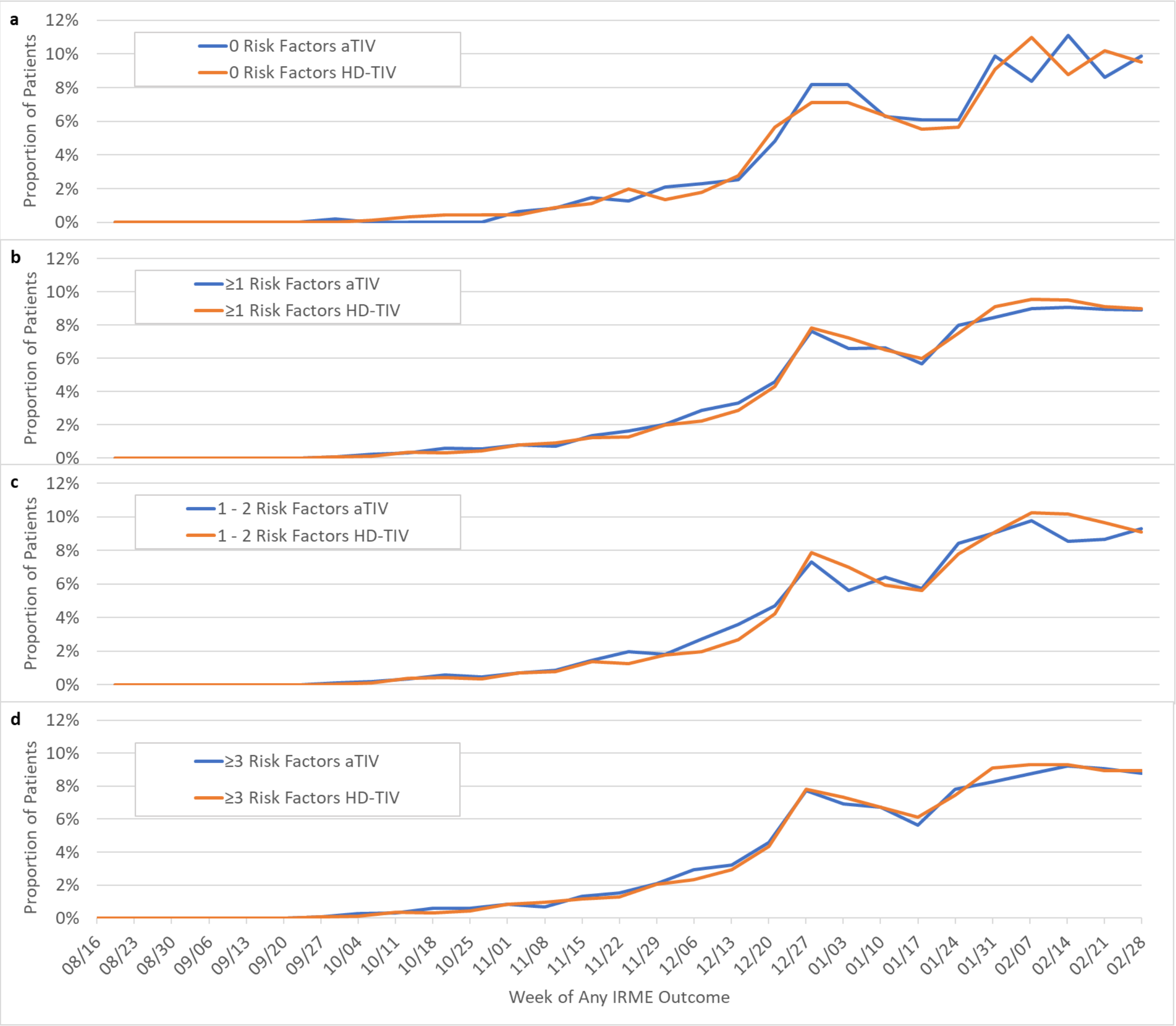

Figure S2. Week of outpatient IRME by vaccine for (a) 0 risk factors, (b) ≥1 Risk Factors, (c) 1–2 Risk Factors, and (d) ≥3 Risk Factors

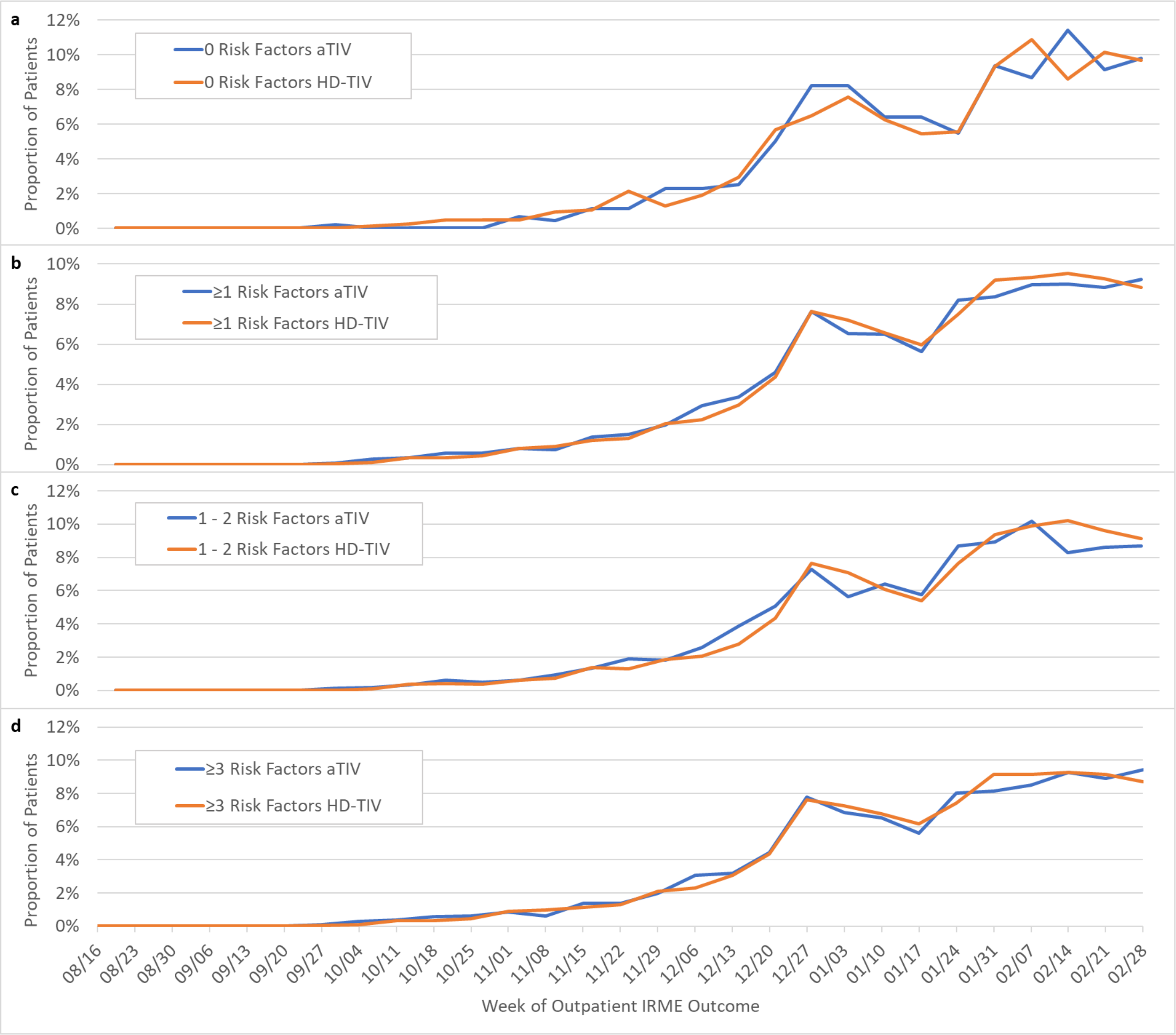

Figure S3. Week of influenza- or pneumonia-related hospitalization by vaccine for (a) 0 risk factors, (b) ≥1 Risk Factors, (c) 1–2 Risk Factors, and (d) ≥3 Risk Factors

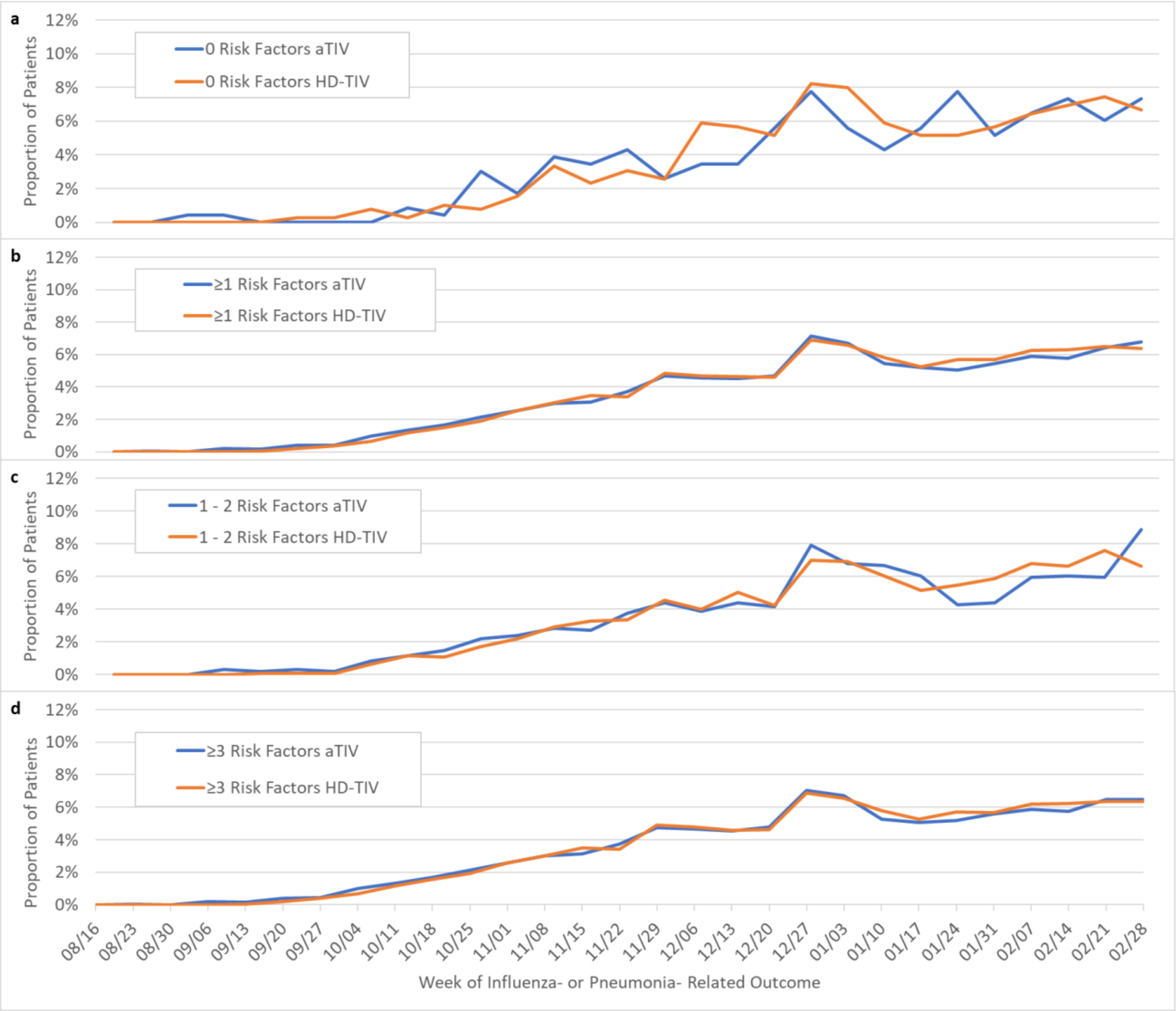

Supplement: ofae459_Supplementary_Data [file ofae459_supplementary_data.pdf]
